# Comment on “accuracy and precision of four main glucometers used in a sub-Saharan African country: a cross-sectional study” by Choukem *et al*

**DOI:** 10.11604/pamj.2019.33.271.19704

**Published:** 2019-07-30

**Authors:** Guido Freckmann, Stefan Pleus, Annette Baumstark

**Affiliations:** 1 Institut für Diabetes-Technologie, Forschungs und Entwicklungsgesellschaft mbH an der Universität Ulm, Ulm, Germany

**Keywords:** Accuracy, glucometer, precision

## Abstract

In their article, Choukem *et al*. report of assessments of the analytical quality of blood glucose monitoring systems. Although there are some commendable aspects regarding the methodology, some major shortcomings could preclude the conclusions drawn by Choukem *et al*. Nevertheless, independent assessments of the performance of blood glucose monitoring systems are an important issue.

## Commentary

In their recent publication, Choukem *et al*. [1] report of accuracy and precision assessments of four different blood glucose monitoring systems (BGMS) or “glucometers”. They concluded that all four BGMS did not fulfill accuracy requirements of ISO 15197. In addition, they reported large positive bias (i.e., BGMS measurement results were found to be systematically higher than reference measurement results). Although there are some commendable aspects regarding the methodology, there are also some major shortcomings that, in our opinion, do not allow for drawing the conclusions as reported by Choukem *et al*. Choukem *et al*. purchased the BGMS from pharmacies, thus using meters and test strips that are representative of those used by end-users. In the study, capillary blood was applied, which typically is the type of sample used by diabetes patients, because they do not readily have access to, for example, venous blood. Rotating the order in which the BGMS are used helps minimizing potential order effects. This shows that the authors were aware of several potential influencing factors and tried to minimize their impact. However, the methods used in the study by Choukem *et al*. had some flaws: Such comparative assessments should use the same sample type for the investigated systems as well as for reference measurements, because glucose concentrations can be different in venous and in capillary blood samples [2]. Furthermore, sodium fluoride/oxalate does not adequately inhibit glycolysis within the first hours [3], so that glucose concentrations would have decreased over the up to 120 minutes passing between sample collection and performance of reference measurements. Last but not least, all reference measurements were performed on a Cobas c111 glucose analyzer, which is based on the hexokinase method. Considering that the CodeFree and OneTouch Ultra are calibrated against a glucose oxidase method, this may also affect the perceived bias. In the literature, as well as our own studies, systematic measurement differences of up to 8% were found between measurement results from glucose oxidase- and hexokinase-based analyzers [4]. In addition, Choukem *et al*. state that requirements of ISO 15197 are not met. However, this statement can only be made with limits because of relevant methodological deviations from the recommended procedure as outlined in ISO 15197 [5]. ISO 15197 requires, for example, that both BGMS and reference measurements are performed in capillary blood. It also requires a specific distribution of glucose concentrations, including 5% of values ≤50 mg/dl and 5% of values >400 mg/dl. Additionally, reference samples are supposed to be drawn before and after BGMS measurements to enable exclusion of samples with unstable glucose concentrations. The accuracy limits of ISO 15197 are misrepresented in the article. With the methods applied by Choukem *et al*., therefore, it is impossible to verify whether BGMS fulfill ISO 15197 accuracy criteria or not.

## Conclusion

These methodological flaws could likely lead to reference measurement results being systematically different than BGMS measurement results. Although their individual contribution to a systematic difference is hard to pinpoint, their combined effect would explain the positive bias values found by Choukem *et al*. at least in part. In addition, some of the requirements regarding methodology as outlined in ISO 15197 were not adhered to by Choukem *et al*. Therefore, it is our opinion that the conclusions drawn by Choukem *et al*. may possibly have been different if these methodological flaws had been avoided. Nevertheless, we want to encourage Choukem *et al*. to perform further testing, as we believe that independent assessment of BGMS performance is an important issue.

## Competing interests

The authors declare no competing interest.
